# Effects of Training with Different Modes of Strength Intervention on Psychosocial Disorders in Adolescents: A Systematic Review and Meta-Analysis

**DOI:** 10.3390/ijerph18189477

**Published:** 2021-09-08

**Authors:** Guillermo Barahona-Fuentes, Álvaro Huerta Ojeda, Luis Chirosa-Ríos

**Affiliations:** 1 Department Physical Education and Sports, Faculty of Sport Sciences, University of Granada, 18011 Granada, Spain; lchirosa@ugr.es; 2 Grupo de Investigación en Salud, Actividad Física y Deporte ISAFYD, Universidad de Las Américas, Sede Viña del Mar 2531098, Chile; achuertao@yahoo.es

**Keywords:** strength training, anxiety, stress, depression, adolescence

## Abstract

Physical exercise has a positive impact on anxiety and depression. However, the evidence that associates strength training with a decrease in adolescents’ psychosocial disorders is scarce. Consequently, the objective was to analyze the effects of training with different modes of strength intervention on anxiety, stress, and depression in adolescents. The search was designed according to PRISMA^®^. We searched WoS, Scopus, SPORTDiscus, PubMed, and MEDLINE (2010–2020). Methodological quality and risk of bias were assessed with the Cochrane Collaboration. The analysis was carried out with a standardized mean difference (SMD) pooled using the Hedges g test (95% CI). The Main Outcome Measures were: anxiety, stress, and depression in adolescents post strength training. Nine studies were included in the systematic review and seven in the meta-analysis. These studies showed a large and significant effect of strength training on anxiety (SMD = −1.75; CI = 95%: −3.03, −0.48; *p* = 0.007) and depression (SMD = −1.61; CI = 95%: −2.54, −0.67, *p* = 0.0007). In conclusion, training with different modes of strength intervention have shown control over anxiety and depression in adolescents. However, conventional strength training seems to have better results than other modes of strength intervention.

## 1. Introduction

Currently, a large part of the population has been affected in terms of quality of life due to negative alterations in mental health [1]. In this sense, variables such as anxiety are considered an emotional reaction consisting of a unique combination of feelings of tension, apprehension, and nervousness, as well as unpleasant thoughts of worry and physiological changes associated with the activation of the autonomic nervous system [2]. Stress is defined as a psychological condition that occurs when a subject observes a substantial imbalance between the demands that he or she endures and his or her ability to meet them [3]. Moreover, depression is defined as a mood condition, such as excessive sadness and/or a significantly reduced experience of pleasure. These mental health disorders are called psychosocial disorders [4,5]. These psychosocial disorders have been identified in athletes [6], workers [1], university students [5,7], and adolescents [8]. Specifically, an adolescent psychosocial disorder can lead to decreased academic performance, increased drug use and consumption, and increased potential for suicidal ideation [8] and has even been associated with decreased interest in physical activity [9]. The latter has been considered one of the determining factors for reducing anxiety, stress, and depression levels [10].

In this context, physical activity and exercise practice improve physical fitness, resulting in beneficial effects on physical and cognitive health in children and adolescents [11]. An example of this is the anxiolytic effect of aerobic exercise [12] in patients with anxiety and stress disorders [13,14], despite this evidence, which shows a positive effect of aerobic physical exercise on anxiety and stress in children and adolescents, leaving a vast space of ignorance about the effect of strength training on psychosocial parameters [15]. Another mechanism by which physical activity and exercise are related to better mental health is brain development in the population that practices physical activity and exercise [16]. In this regard, it has been shown that high levels of maximum oxygen consumption are related to a greater volume of the left medial prefrontal cortex and an increase in the surface areas of the parietal cortex in adolescents [17]. In parallel, it has been found that subjects with a better physical shape have higher hippocampus volumes [18]. Conversely, a lower hippocampus volume has also been associated with the development and increased depression levels in adolescents [19,20,21,22].

As mentioned above, the practice of physical activity and exercise may be involved in brain development [16] and, consequently, in psychosocial disorders [23]. Although the benefits of aerobic training on anxiety, stress, and depression are well established in the literature [14,15], evidence from strength training on psychosocial disorders is scarce [24]. Specifically, strength training is an exercise that involves the voluntary activation of specific muscle groups of the skeletal muscle against an external resistance [25]. There is training with different modes of strength intervention that allow the development of muscular strength: conventional strength training (which consists of systematic executions of concentric and eccentric muscle contractions voluntarily against external loads) [26]; concurrent training (which includes a combination of aerobic and strength training) [27]; strength training on a vibrating platform (which includes the use of vibrations to induce muscle contraction) [28]; strength training through CrossFit^®^ (which consists of a combination of strength exercises performed through a circuit) [29], and plyometric training (which are jumping exercises) [30]. This training type allows the development and increase of skeletal muscle mass, strength, power, and muscle endurance [31,32,33]. Concerning optimal muscle development resulting from strength training, it has been shown that subjects with greater muscle mass have a better quality of life [34]. Conversely, low levels of muscle strength are a reflection of poor muscle quality [35]. In this sense, muscle quality describes the functional physiological capacity of muscle tissue [36]. This tissue must carry out various functions, such as contractions, metabolism, and electrical conduction [36]. At the same time, it has been observed that high muscle quality plays a role in preventing chronic diseases [37]. It has also been shown that men and women with a high subcutaneous adipose tissue content [38], high-fat mass, and low lean mass [39] have decreased muscle quality [38,39].

Based on these antecedents, it is possible that an excellent physical condition—reflected in good muscular quality as a result of strength training [35]—might be capable of making substantial changes in brain development [16]. This may decrease anxiety and stress levels, as well as depression in adolescents. Unfortunately, these associations are not sufficiently studied; so far, it seems that only speculations were made. On the other hand, there would be indications that strength training would improve anxiety levels [24]. In this regard, a meta-analysis by Gordon et al. [24] showed that strength training significantly improved anxiety symptoms in healthy adult participants and adult participants with a physical or mental illness. However, these researchers [24] did not evaluate the anxiety, stress, and/or depression of the adolescent participants; thus, there is insufficient knowledge about the effects of training methods on adolescents’ anxiety, stress, and depression levels.

Consequently, the objective of this systematic review and meta-analysis was to analyze the effects of training with different modes of strength intervention on psychosocial disorders of anxiety, stress, and depression in adolescents.

## 2. Materials and Methods

This systematic review and meta-analysis followed the preferred reporting items for systematic reviews and meta-analyses (PRISMA) guidelines [40] and the Cochrane Collaboration guidelines to evaluate the risk of studies bias. The protocol of this review was registered in PROSPERO (CRD42021271440)

### 2.1. Eligibility Criteria

Articles were eligible if they were published or in-press in a peer-reviewed journal, with full text in English, Spanish, French, Portuguese, and German. Search limits were articles published in the last ten years (January 2010 to June 2020). Such restriction has the objective to show a current panorama of the analyzed studies [41,42]. The literature search was conducted following the guidelines for systematic reviews and meta-analysis (PRISMA) [40]. For this purpose, population, intervention, comparators, and outcomes (PICO) were established as follows: (i) participants were adolescents explicitly or implicitly diagnosed with any form of anxiety, depression, and stress (for example, Beck’s Depression Inventory (BDI) [43], Children’s Depression Inventory (CDI) [44], Profile of Mood States (POMS) [45], Premenstrual Symptoms Impact Survey (PMSIS) [46], State-trait Anxiety Inventory (STAI) [47], Children’s Manifest Anxiety Scale (CMAS) [48], Kessler Psychological Distress Scale (K10) [49], among others [50]); (ii) Only those interventions that, within their protocol, have used different modes of strength training intervention, regardless of their modality (alone or combined), were considered; (iii) The comparators were control groups that had not performed any training protocols; (iv) The results were any effects (positive or negative) on indicators of anxiety, stress, and depression; (v) The study design was limited to experimental studies. Studies that did not meet the eligibility criteria were excluded. The discrepancies found were resolved by the consensus of the researchers.

### 2.2. Information Sources and Search

The search identified articles published in the following databases: Web of Science (WoS), Scopus, SPORTDiscus, PubMed, and Medline. In each of the databases, the title, abstract, and keyword search fields were searched. The following keywords, combined with Boolean operators (AND/OR), were used ([“strength training” OR “resistance training” OR “weight training” OR “concurrent training” OR “combined training” OR “cross training” OR “crossFit” OR “plyometric training”] AND [“adolescent” OR “adolescence” OR “teenager” OR “teen”] AND [“anxiety” OR “stress” OR “depression” OR “depressive disorder”]). Two authors searched and reviewed the studies, both deciding whether the inclusion of the studies was appropriate. In case of disagreement, a third author was consulted.

### 2.3. Data Extraction

The data collection was: author, year, journal, target, sample, number of participants, age, dependent and independent variable, treatment, outcomes, performance, experimental, and control groups. One author extracted the continuous data for the meta-analysis and a second author verified them. In case of disagreement, the third author was consulted. The values were entered in a spreadsheet in the Excel software, and then the Review Manager software was used (version 5.4) (Copenhagen, Denmark: The Nordic Cochrane Centre, The Cochrane Collaboration, 2014).

### 2.4. Risk of Publication Bias between Studies

The risk of publication bias between studies was only carried out in those part of the meta-analysis. Publication bias was assessed using Egger’s statistical test. This test determined the presence of bias at *p* ≤ 0.05 [51]. Funnel plots were created to interpret the general effect, followed by an Egger’s statistic to confirm or refute publication bias.

### 2.5. Methodological Quality and Risk of Bias of Individual Studies

The methodological quality and risk of bias of each study selected for the meta-analysis were evaluated using the Cochrane Collaboration guide [52]. The list was divided into six different domains: selection bias (random sequence generation, allocation concealment), performance bias (blinding of participants and personnel), detection bias (blinding of outcome assessment), attrition bias (incomplete outcome data), reporting bias (selective reporting), and other types of bias (declaration of conflict of interest). For each item, the answer to a question was considered; when the question was answered with a “Yes,” the bias was low; when it was “No,” the bias was high; when it was “Unclear,” the possible bias was connected to a lack of information or uncertainty.

### 2.6. Summary Measures and Synthesis of Results in Studies

For the analysis and interpretation of results in this systematic review and meta-analysis, the effect of strength training on anxiety, stress, and depression levels in adolescents was examined as a primary outcome. The meta-analysis was only carried out if the selected study complied with an intervention with a strength training protocol, contained a control group and an experimental group, and those in which the variables of anxiety, stress, and/or depression had presented pre- and post-intervention evaluations. Thus, if any study did not meet these characteristics, it could not be part of the meta-analysis and would only be considered part of the systematic review. In order to evaluate the quality of the experiments and interpret the risk of bias values, Review Manager version 5.4 was used (Copenhagen, Denmark: The Nordic Cochrane Centre, The Cochrane Collaboration, 2014). The same software was used to perform a descriptive and statistical analysis of the meta-analysis. To compare the effects of the experimental group (EG) that performed resistance training versus a control group (CG) that contained no intervention, the number of participants, standardized mean difference (SMD), and standard error of SMD were analyzed for each study. Hedges’ g test was used to calculate the SMD of each study [53]. The overall effect and the 95% confidence interval (CI) were calculated by weighting the SMD by inverse variance. Additionally, the SMD of both the EG and CG groups were subtracted to obtain the effect size (ES), which was used together with the pooled SD of change to calculate the variance (ES = [mean EG − mean CG]/SD). To interpret the magnitude of the ES, Cohen’s criteria were: <0.2, trivial; 0.2–0.5, small; 0.5–0.8, moderate; and >0.8, large [54].

Due to real heterogeneity rather than chance, the I2 statistic was calculated as an indicator of the studies’ total observed variation. I2 values are included from 0 to 100%, representing: a small amount of inconsistency (between 25% and 50%); a medium amount of heterogeneity (between 50% and 75%); and a large heterogeneity (when the I2 value was higher than 75%). In this sense, low, moderate, and high adjectives would be accepted, referring to I2 values of 25%, 50%, and 75%, respectively, although a restrictive categorization would not be adequate in all circumstances [55].

## 3. Results

### 3.1. Studies Selection

**Figure 1:**
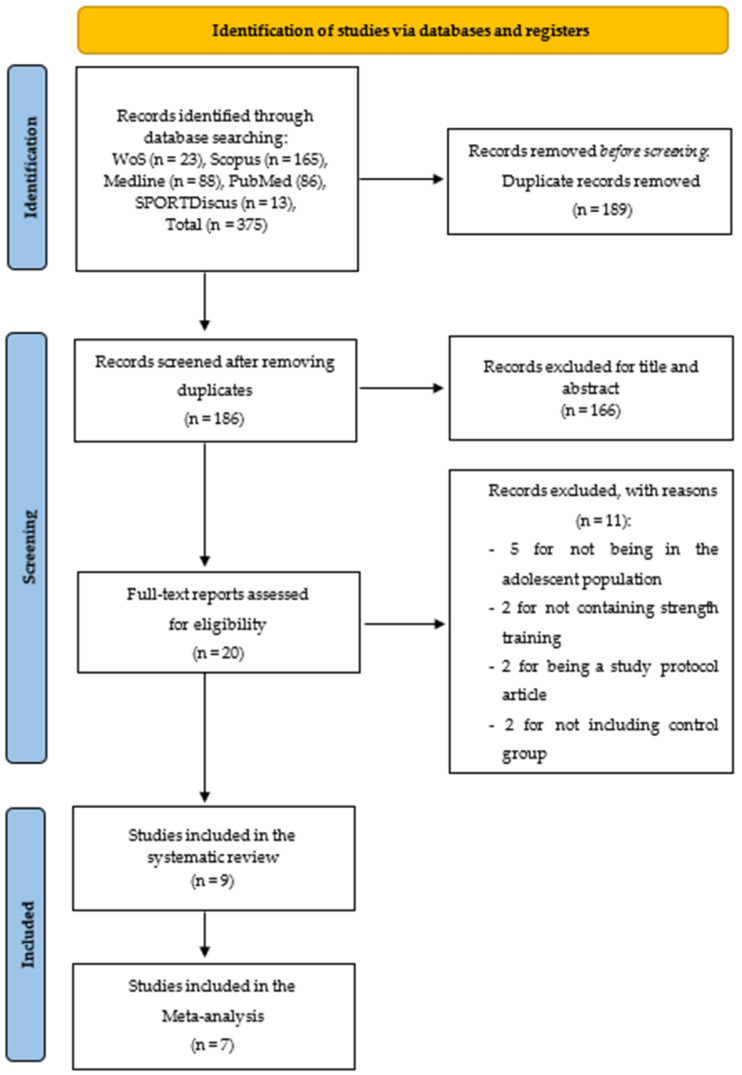
PRISMA flow diagram of articles that were selected.

The literature search through electronic databases identified 375 articles, of which 189 were duplicates. The remaining 166 articles were filtered by title and abstract, and 20 studies remained to be read and analyzed. After reviewing those 20 studies, 11 were eliminated because they did not meet the inclusion criteria. As a result, nine articles were included in the systematic review. Of these nine, two did not meet the meta-analysis characteristics, thus only seven studies were part of the meta-analysis. The search strategy and study selection are shown in Figure 1.

Of the nine studies included in the systematic review and meta-analysis, one determined the effects of strength training on anxiety levels [56], four on depression [57,58,59,60], and three used anxiety and depression variables together [61,62,63]. Besides, one article evaluated mental health (emotional, psychological, and social well-being) [64], which is commonly associated with psychosocial anxiety disorders and depression [65]. On the other hand, strength training was reflected through the following intervention modes: CrossFit Teens™ [64], concurrent training (CT) combining aerobic and strength exercises [56,57,58,61,63], vibration platform strength training [60,62], and conventional strength training [58,59,62]. The characteristics and type of strength training protocol of the studies selected in this systematic review and meta-analysis are presented in Table 1 and Table 2.

#### 3.1.1. Risk of Bias among Studies

**Figure 2:**
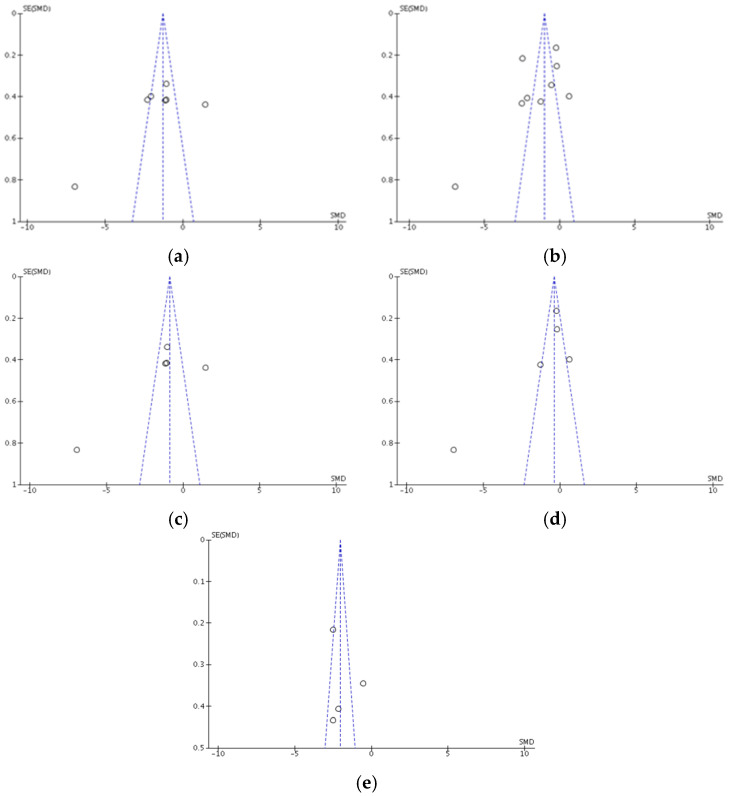
The standard error in different modes of strength intervention for anxiety and depression. (**a**) strength training and concurrent training for anxiety; (**b**) strength training and concurrent training for depression; (**c**) concurrent training for anxiety; (**d**) concurrent training for depression; (**e**) strength training for depression; SE: standard error; SMD: standardized mean difference.

Egger’s analysis suggested that the primary variables evaluated in the studies that were part of the meta-analysis showed publication bias after strength training and concurrent training: (a) anxiety: z = 2.69, *p* = 0.007; (b) depression: z = 3.38, *p* = 0.0007 (Figure 2).

#### 3.1.2. Assessment of Methodological Quality and Risk of Bias of Individual Studies

**Figure 3:**
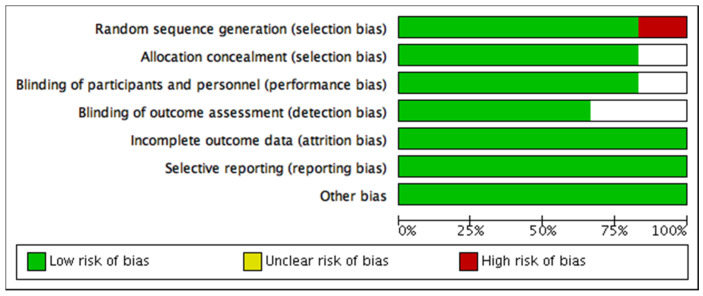
Risk of bias graph: review authors’ judgements about each risk of bias item presented as percentages across all included studies.

**Figure 4:**
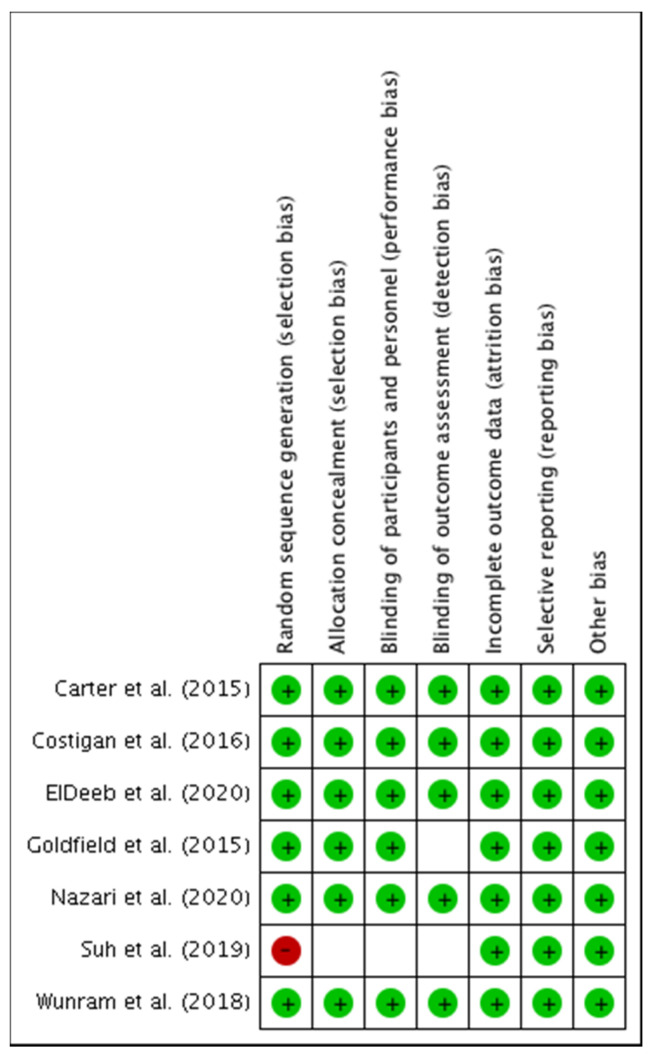
Risk of bias summary: Review authors’ judgements about each risk of bias item for each included study.

The assessment of the methodological quality and risk of bias of the seven studies selected for meta-analysis showed that the study developed by Suh et al. [63] had: a high risk of bias for the domain of selection bias (random sequence generation, allocation concealment); unclear risk for performance bias (blinding of participants and research staff); detection bias (blinding of outcome assessment); and dropout bias (incomplete outcome data). Likewise, Goldfield et al. [58] showed an unclear risk of bias for dropout bias. On the other hand, the rest of the studies [56,57,60,61,62] showed a low risk of bias for all domains. Full details of each study and domain are presented in Figure 3 and Figure 4.

### 3.2. Meta-Analysis

#### 3.2.1. Effects of Different Strength Training Methods on Anxiety Levels

**Figure 5:**
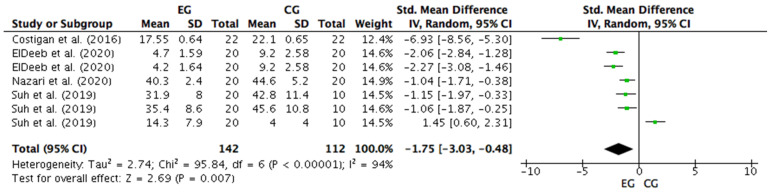
Forest plot comparing the effects of training with different modes of strength intervention on anxiety levels. EG: experimental group; CG: control group; SD: standard deviations.

Four studies were considered for this analysis [56,61,62,63]. However, Suh et al. [63] included three anxiety questionnaires in the research with different results. On the other hand, ElDeeb et al. [62] included two different pieces of training for strength intervention in the research design, one on a vibrating platform and the other conventional. For the meta-analysis, the study by Suh et al. [63] was considered as three independent designs. Similarly, the study by ElDeeb et al. [62] was considered as two independent training protocols. Therefore, seven studies were included in the meta-analysis that calculated the effect of training with different modes of strength intervention on anxiety levels. Figure 5 shows that training with different modes of strength intervention have a large and significant effect on the anxiety level (SMD = −1.75; CI = 95%: −3.03, −0.48; *p* = 0.007). The meta-analysis showed high heterogeneity among the studies reviewed (I2 = 94%; *p* = 0.00001). Out of the seven studies analyzed, six reported a beneficial effect of different strength training methods on anxiety levels [56,61,62,63]. These six studies showed a large ES in anxiety levels: ES = −6.93 through the K10 after concurrent training [61]; ES = −2.06 through the PMSIS on both the group that received strength training on the vibration platform and the group that performed conventional strength training [62]; ES = −1.04 through the CMAS for the group undergoing concurrent training [56]; and ES = 1.15 and ES = 1.06 through the STAI for trait and state, respectively, in a group that carried out concurrent training [63]. However, the research of Costigan et al. [61] presented a higher ES (−6.93) than the rest. On the other hand, one of these seven studies showed no effect after strength and concurrent training on anxiety levels measured by the CMAS (ES = 1.45) [63].

#### 3.2.2. Effects of Training with Different Modes of Strength Intervention on Depression Levels

**Figure 6:**
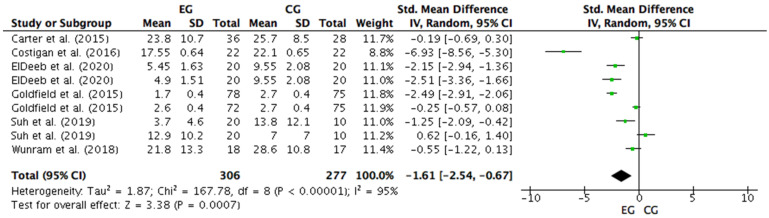
Forest Plot comparing the effects of training with different modes of strength intervention on depression levels. EG: experimental group; CG: control group; SD: standard deviations.

Six studies were considered for this analysis [57,58,60,61,62,63]. However, ElDeeb et al. [62] included two different pieces of training for strength intervention, one on a vibrating platform and another conventional strength training in the research design. Thus, for the meta-analysis, ElDeeb et al.’s [62] study was considered as two independent training protocols. Goldfield et al. [58] used two different pieces of training for strength intervention, one conventional strength training, and another concurrent training that combined aerobic and strength exercises. Thus, the study by Goldfield et al. [58] was also considered as two independent training protocols. On the other hand, Suh et al. [63] included two depression questionnaires in the research with different results. Thus, Suh et al.’s [63] study was considered as two independent designs for the meta-analysis. Therefore, nine studies were considered in the meta-analysis that calculated the effects of training with different modes of strength intervention on depression levels. Figure 6 shows that training with different modes of strength intervention have a large and significant effect on the anxiety level (SMD = −1.61; CI = 95%: −2.54, −0.67; *p* = 0.0007). The meta-analysis showed high heterogeneity among the studies reviewed (I2 = 95%; *p* = 0.00001). Of the nine studies analyzed, eight of them reported a beneficial effect of training with different modes of strength intervention on depression levels [57,58,60,61,62,63]. Of these eight studies, five showed a large ES in the levels of depression. They measured ES = −6.93 through K10 [61]; ES = −2.15 and ES = −2.51 through the PMSIS in a group that received strength training on a vibrating platform, and the group that performed conventional strength training [62]; ES = −2.49 through the Brunel Mood Scale (BRUMS) for the group undergoing conventional strength training [58]; and ES = −1.25 through the BDI [63]. However, research by Costigan et al. [61] presented a higher ES (−6.93) over the rest. On the other hand, one study showed a moderate ES (−0.55) through the Child and Adolescent Depression Inventory (DIKJ) after strength training on the vibration platform [60]. A study had a small ES (−0.25) in depression through the BRUMS for the group undergoing concurrent training [58]. Finally, out of the eight studies that reported beneficial effects on depression levels after strength training and concurrent intervention, only one obtained a trivial ES (−0.19) in depression through the Children’s Depression Inventory 2nd Version (CDI-2), following concurrent training [57]. On the other hand, a study that measured depression through the CDI showed no effect after the conventional strength training intervention (ES = 0.62) [63].

#### 3.2.3. Effects of Concurrent Training on Depression

**Figure 7:**
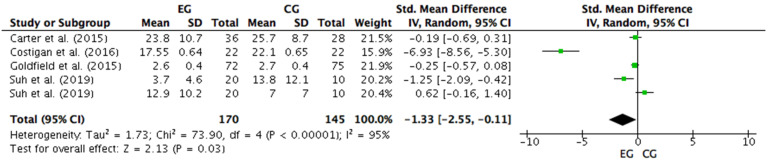
Forest plot comparing the effects of concurrent training on depression levels. EG: experimental group; CG: control group; SD: standard deviations.

Four studies were considered for this analysis [57,58,61,63]. However, Suh et al. [63] included two depression questionnaires in the research with different results. Thus, Suh et al.’s [63] study was considered as two independent designs for the meta-analysis. Therefore, five studies were considered in the meta-analysis that calculated the effect of concurrent training on depression levels. Figure 7 shows that concurrent training generates a large and significant effect on the anxiety level (SMD = −1.33; CI = 95%: −2.55, −0.11; *p* = 0.03). The meta-analysis showed high heterogeneity among the studies reviewed (I2 = 95%; *p* = 0.00001). Out of the five studies analyzed, four of them reported a beneficial effect of concurrent training on depression levels [57,58,61,63]. Out of these four studies, two of them showed a large ES in levels of depression measured by the K10 (ES = −6.93) [61] and the CDI (ES = −1.25) [63]. However, the study by Costigan et al. [61] presented a higher ES (−6.93) above that of Suh et al. [63]. A study also showed a small ES (−0.25) through the BRUMS [58]. Finally, out of the four studies that reported beneficial effects on depression levels after the concurrent training intervention, only one obtained a trivial ES (−0.19) in depression through the CDI, following concurrent training [57]. On the other hand, a study that measured depression through the CDI showed no effect after the concurrent training intervention (ES = 0.62) [63].

#### 3.2.4. Effects of Conventional and Vibration Platform Strength Training on Depression

**Figure 8:**
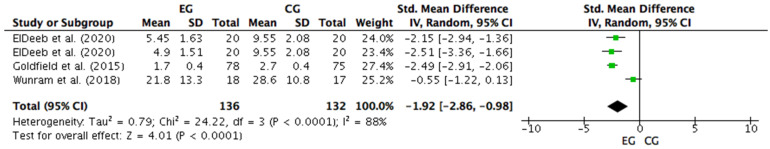
Forest plot comparing the effects of conventional and vibration platform strength training on depression levels. EG: experimental group; CG: control group; SD: standard deviations.

Three studies were considered for this analysis [58,60,62]. However, ElDeeb et al. [62] included two different pieces of training for strength intervention, one conventional and another on a vibrating platform. Therefore, for the meta-analysis, ElDeeb et al.’s [62] study was considered as two independent training protocols. Therefore, four studies were considered in the meta-analysis that calculated the effect of strength training on depression levels. Figure 8 shows that strength training generates a large and significant effect on the level of depression (SMD = −1.92; CI = 95%: −2.86, −0.98; *p* = 0.0001). The meta-analysis showed high heterogeneity among the studies reviewed (I2 = 88%; *p* = 0.0001). The four studies analyzed declared a beneficial effect of strength training on depression levels [58,60,62]. Out of these four studies, three showed a large ES in the levels of depression. The ES was measured through the PMSIS in both the group that received vibration platform strength training (ES = −2.15) and the group that performed conventional strength training (ES = −2.51) [62], and through the BRUMS in the group that underwent conventional strength training (ES = −2.49) [58]. A study also showed moderate ES through the DIKJ after strength training on the vibration platform (ES = −0.55) [60].

## 4. Discussion

Regarding the studies included in the systematic review and meta-analysis, the results showed that concurrent training [56,57,58,61,63], conventional strength training [58,59,62], vibration platform strength training [60,62], and strength training through Crossfit™ Teens [64] had been used to control or decrease the levels of anxiety and depression. Thus, it was possible to demonstrate that physical exercise, through strength training independent of its modality, produces decreases in the levels of anxiety (ES = −1.75) and depression (ES = −1.61) in 448 adolescent subjects (142 and 306, respectively). Other meta-analyses indicated a small to moderate effect for anxiety [24] and depression [66]. These studies in the adult population determined the effects of strength training on anxiety and depression [24,66]. However they included only randomized controlled studies and excluded those studies that combined strength training with another modality, such as concurrent training and vibration platform strength training [24,66]. This differs from our meta-analysis, which showed a large effect after the training with different modes of strength intervention on anxiety and depression levels in the adolescent population. These differences may be a product of the level of neuroplasticity that exists in adolescents over adults [67], which would allow further modeling of brain development [22] and therefore provide more encouraging benefits for adolescent mental health [67].

### 4.1. Physical Performance and Psychosocial Disorders of Anxiety, Stress, and Depression

At the end of this systematic review, the only physical performance effects reported and associated with strength training were maximum strength and maximum oxygen consumption. However, only three studies evaluated these variables [59,60,63], and only two of these studies showed an increase in physical performance for the groups undergoing strength training [59,63]. In this sense, Gordon et al. [59] intervened in a group with AT and another with ST, showing a decrease in depression variables in both groups. However, the same authors showed an increase (*p* = 0.05) in push-up for the ST group as opposed to the AT group (*p* > 0.05). Likewise, there is evidence that physical exercise through strength training produces different physiological adaptations, such as increased muscle mass, muscle strength, and muscle power [32,33]. These three concepts together are strongly associated with muscle quality [36], which refers to the ability of skeletal muscles to perform several functions effectively, including force production, contraction and relaxation, metabolism, substrate turnover and storage, heat generation, myokine production, and electrical conduction [36]. In this context, the literature has described that low muscle strength levels may reflect weak muscle quality [35], while high muscle quality can help prevent chronic disease [37]. It has also been associated that adolescents who present some chronic diseases have a higher level of anxiety and depressive symptoms [68]. Thus, a possible association between muscle strength and quality with psychosocial disorders could be increased hippocampus and some markers’ activation. However, this has not been established; so far, it would only be speculation.

### 4.2. Effects of Different Methods of Strength Training Associated with Psychosocial Disorders of Anxiety, Stress, and Depression

Specifically, strength training through a concurrent method has been the most used to mitigate or decrease anxiety and depression in adolescents [56,57,58,61,63]. In this sense, Wegner et al. [15] showed that the meta-analyses that have studied the effect of physical exercise on depression are mainly studies involving aerobic exercise and not strength training. In this sense, the evidence indicates that aerobic exercise is a favorable alternative for reducing anxiety and depression in children and adolescents [15]. However, the ES (>0.8) obtained in the present meta-analysis suggests that strength training, regardless of its modality, is a good alternative for controlling psychosocial disorders in adolescents [54]. This meta-analysis has shown that both strength training—conventional and through a concurrent—methods present a large ES in the levels of depression. When performing a subdivision of the training methods and determining their effects on depression, concurrent training evidence of an ES = 1.33 besides a strength training evidence of an ES = 1.92 may indicate more significant depression benefits through conventional strength training over concurrent training. The causes may be the possible physiological interferences that aerobic training would cause on the hypertrophy and muscular strength induced by strength training [69]. Scientific evidence shows that high levels of muscle hypertrophy and strength are stimulated by anabolic hormones such as growth hormone (GH) [70,71] and possibly by insulin-like growth factor-1 (IGF-1) [72]. In this context, it has been described that GH and IGF-1 would have a fundamental role in the growth and maintenance of the central nervous system and the peripheral nervous system [73]. However, other studies [74,75,76] have shown that high values of IGF-1 are associated with higher levels of depression. Likewise, a recent meta-analysis showed that higher levels of IGF-1 are found in aerobic exercise over strength training [77]. This background may partly explain why there are more significant benefits for depression through conventional strength training than through concurrent and aerobic training. However, this history should be taken with caution because the role of IGF-1 is not conclusive for the treatment and diagnosis of depression [75,78]. On the other hand, due to the low amount of scientific information, we could not compare the effect of concurrent training and strength training on anxiety levels because there were more studies with concurrent training [56,61,63] over strength training [62].

### 4.3. Strength Training and Brain Development Associated with Psychosocial Disorders of Anxiety, Stress, and Depression

Simultaneously, the meta-analysis showed that strength training, regardless of its modality, presents no evidence of stress levels in adolescents [56,57,58,59,60,61,62,63,64]. In this sense, Nazari et al. [56] evaluated cortisol levels as a sign of stress [79]; however, in our meta-analysis, it has not been possible to establish this association as cortisol would not be a reliable measure in adolescents [67]. In connection with this, Wu et al. [67] concluded that perceived stress through questionnaires is a more sensitive indicator than cortisol measurement for reflecting emotional states and diagnosing stress levels in adolescents, mainly due to neuroplasticity developmental factors present in adolescents’ brains [22,67]. Stress has also been associated with symptoms of anxiety and depression [80]. On the other hand, depression has been associated with stress and reduced hippocampus [81]. In this sense, some studies have found an association between a lower hippocampus volume with more significant depressive symptoms [19,21]. Moreover, the hippocampus volume is related to the severity of depressive symptoms and the duration of the illness [82]. Additionally, the literature has described that sedentary behavior may have the potential to negatively influence the brain structure of overweight or obese children [83]. In contrast, physical exercise may have important implications for brain development [16] and, therefore, in psychosocial disorders [23]. Thus, in a study by Feter et al. [84], brain adaptations were evidenced by the increase of the hippocampus volume due to physical exercise. Moreover, there are indications that strength training can generate positive responses in the volume of the hippocampus and an increase in the concentration of IGF-1, which could play an essential role in the creation and protection of neurons [85,86], thus favoring a possible control of psychosocial disorders [23]. However, recently Troyan and Levada [76] showed that patients diagnosed with depressive disorders had higher levels of IGF-1, but lower levels of brain-derived neurotrophic factor (BDNF). Similarly, research conducted on subjects who had died by suicide showed a decrease in BDNF mRNA expression compared to control subjects [87]. At the same time, there is evidence to suggest that antidepressant medications, exercise, and strength training can increase BDNF [88,89,90,91], and therefore, the volume of the hippocampus [92] associated with a decrease in psychosocial disorders [19,21]. However, studies on the effects of BDNF and other mediators on brain plasticity [93], protection of the hippocampus, and plausible neurobiological adaptations to strength training are lacking [24,84], as well as the role that IGF-1 would have on psychosocial parameters [74,75,76,85,86] since IGF-1 remains ambiguous for the treatment and diagnosis of depression [75,78] over other markers [94]. Unfortunately, it has not been possible for the meta-analysis to prove these associations between brain and strength training reliably. Despite this, we believe and support the recent research by Gorham et al. [23], who explained that a reduction in psychosocial disorders associated with sports participation may be related to a neural mechanism, because physical exercise would cause an increase in the volume of the hippocampus. However, like these authors [23], we believe that more research is needed to understand the causal relationships between these variables.

## 5. Conclusions

There are indications that different modes of strength intervention are a suitable methodology for controlling anxiety and depression levels in adolescents. Specifically, our meta-analysis indicates that conventional strength training has better benefits than other modes of strength intervention. However, this field has not been investigated in-depth, thus further experimental studies focusing on strength training to control or mitigate anxiety, stress, and depression levels in the adolescent population are needed. This will allow new public policies and programs to assess, control, and mitigate psychosocial disorders through training that features different modes of strength intervention.

## Figures and Tables

**Table 1:** Characteristics of the studies included in the systematic review and meta-analysis.

Author	Objective	Sample	Variables	Results	Physical Performance
Effects of Strength and Concurrent Training on Anxiety and Depression					
Costigan et al. [61]	To evaluate the efficacy of two high-intensity interval training (HIIT) protocols for improving cognitive and mental health outcomes (executive function, psychological well-being, psychological distress, and physical self-concept) in adolescents	65 adolescents, 45 M and 20 W CG: 22 (15.6 ± 0.6) EG1: 21 (15.7 ± 0.7) EG2: 22 (15.5 ± 0.6)	IV: HIIT (EG1); HIIT + ST (EG2) DV: Cognitive and mental health (evaluated through the Kessler Psychological Distress Scale).	ns	--
Eather et al. [64]	To investigate the effectiveness of the CrossFit Teens resistance training program for improving mental health outcomes in adolescents and to explore potential moderators and mediators.	96 adolescents (15.5 ± 0.50) CG: 45 EG: 51	IV: CrossFit Teens. DV: Mental health (evaluated through the Strength and Difficulties Questionnaire)	ns	--
Suh et al. [63]	To evaluate the effect of combined aerobic and resistance exercise in adolescents with type l diabetic.	30 adolescents with Diabetes Mellitus l EG: 20 (17.10 ± 4.54) CG: 10 (21.80 ± 3.05)	IV: CT. DV: Anxiety, depression (evaluated through the Beck’s Depression Inventory, Children’s Depression Inventory, State-trait Anxiety Inventory and Revised Children’s Manifest Anxiety Scale). Glycemic control, cardiovascular function, and physical fitness	EG ↑ (*p* < 0.05) in Vo_2_ max and maximal force. Anxiety and depression were ns.	↑ in both groups
ElDeeb et al. [62]	To compare the effect of whole-body vibration and resistive exercise on premenstrual symptoms in adolescents with premenstrual syndrome.	60 young sedentary W CG: 20 (17.9 ± 1.16) EG1: 20 (17.7 ± 1.17) EG2: 20 (17.3 ± 1.41)	IV: VPT (EG1); ST (EG2). DV: Premenstrual symptoms, anxiety, and depression (evaluated through the Premenstrual Symptoms Impact Survey)	EG1 and EG2, ↓ (*p* < 0.05) their levels of anxiety and depression, significantly.	--
Effects of Strength and Concurrent Training on Anxiety					
Nazari et al. [56]	To explore the effect of concurrent resistance-aerobic training on serum cortisol level, anxiety, and quality of life among pediatric type l diabetic.	40 children adolescents with type 1 diabetes. EG: 20 (11.22 ± 1.90) CG: 20 (11.00 ± 2.67)	IV: CT. DV: Anxiety, serum cortisol level, and quality of life (evaluated through the Revised Children’s Manifest Anxiety Scale).	EG ↓ (*p* = 0.001) anxiety, significantly. Quality of life ↑ significantly (*p* = 0.003). Cortisol was ns.	--
Effects of Strength and Concurrent Training on Depression					
Goldfield et al. [58]	To determine the effects of aerobic training, resistance training, and combined training on mood, body image, and self-esteem in adolescents with obesity.	304 adolescents with obesity. 91 M y 213 W CG: 76 (15.6 ± 1.3) EG1: 75 (15.5 ± 1.4) EG2: 78 (15.9 ± 1.5) EG3: 75 (15.5 ± 1.3)	IV: AT (EG1); ST (EG2); CT [AT + ST] (EG3) DV: Mood with depression, fatigue and anger (evaluated through the Brunel Mood Scale), body image and self-esteem.	EG2 ↓ (p 0.02) their depression levels, significantly.	--
Carter et al. [57]	To determine the effectiveness of a preferred intensity exercise intervention on the depressive symptoms of adolescents with depression.	87 youngsters with depression. 9 M y 68 W CG: 43 (15.4 ± 0.9) EG: 44 (15.4 ± 1.0)	IV: CT. DV: Depression (evaluated through the Children’s depression inventory 2), and quality of life	ns	--
Gordon et al. [59]	To investigate the differential effects of graded aerobic exercise and progressive resistance training on exercise tolerance, fatigue, and quality of life in adolescent patients with chronic fatigue syndrome.	22 adolescents with chronic fatigue. EG1: 11 (16.2 ± 0.8) EG2: 11 (15.6 ± 1.6)	IV: AT (EG1); ST (EG2). DV: Exercise tolerance, fatigue, and depression (evaluated through the Becks Depression Index).	EG1 and EG2 ↓ (p 0.02 and p 0.03) depression levels significantly.	↑ in both groups
Wunram et al. [60]	To investigate the feasibility and effectiveness of a high-frequency whole-body vibration (WBV) training as add-on anti-depressive treatment in medication-naive inpatient adolescents with diagnosed major depression compared to an endurance cycling condition.	64 teenagers with depression. EG1: 20 (16.1 ± 1.2) EG2: 21 (15.9 ± 1.2) CG: 23 (15.7 ± 1.1)	IV: Ergometer (EG1); VPT (EG2). DV: Depressive symptoms (evaluated through the Depressionsinventar für Kinder und Jugendliche).	Depression was ns after 6w. Depression ↓ after 26w EG1 (*p* = 0.037) y EG2 (*p* = 0.042), significantly.	EG1: ↑ EG2: =

↓: decreases; =: equal; ↑: increase; --: not measured; +: plus; AT: aerobic training; CG: control group; CT: concurrent training; DV: dependent variable; EG: experimental group; HR: heart rate; IV: independent variable; M: men; min: minutes; MR: maximum repetitions; ns: non-significant; P: pause; R: repetitions; S: sessions; s: seconds; ST: strength training; VO_2_ max: maximum oxygen consumption; VPT: vibration platform training; W: women; w: weeks; Wo: work; x: for.

**Table 2:** Characteristics of Strength Training Interventions.

Author	W	S/w	Methodology	Reps (n)	Sets (n)	Intensity/Load	Rest Between Sets
Costigan et al. [61]	8	3	AT HIIT (EG1); Gross motor cardiorespiratory exercises (shuttle runs, jumping jacks, and skipping)	Maximum number of repetitions in 30 s for 8–10 min	NR	92.4% (HR max)	30 s
	8	3	CT [HIIT + ST] (EG2); (shuttle runs, jumping jacks, skipping, combined with body weight squats, push-ups)	Maximum number of repetitions in 30 s for 8–10 min	NR	91.8% (HR max)	30 s
Eather et al. [64]	8	2	CrossFit (squat jumps, lunges, medicine ball toss, push-ups, deadlifts and shoulder press)	Depending on the performance obtained W previous	NR	NR	NR
Suh et al. [63]	12	1	CT [AT + ST] (10 min of leg extension and leg press, and 40 min of cycling and walking on the treadmill)	ST = 12 AT = 1	ST = 5 AT = 1	ST = 70% (1-MR) AT = 70–80% (HR max)	NR
ElDeeb et al. [62]	12	3	VPT (with a knee angle of 150° and vibration amplitude of 1 mm).	1-min	3–10	20 Hz	1-min
	12	3	ST (exercises for shoulder, elbow, hip, and knee joints).	3–12	1 for shoulder	60–70% (1-MR)	2-min
Nazari et al. [56]	16	3	CT [ST + AT] (20-min Pilates exercises + 20-min bodyweight exercises. Then, 20-min AT including 10-min of V-forward, V-back and 10-min of march)	ST = 8–12	ST = 2–3	ST = NR AT = 50–75% (HR max)	ST = 30 s AT = 2-min
Goldfield et al. [58]	22	3	AT (EG1); (45-min of Treadmill, elliptical, and/or bicycle)	1	1	65–85% (HR max)	NR
	22	3	ST (EG2); (Seven exercises with weight machines or free weights)	8–15	2–3	80% (1-MR)	NR
	22	3	CT [AT + ST] (EG3)	AT = 1 ST = 8–15	AT = 1 ST = 2–3	AT = 65–85% (HR max) ST = 80% (1-MR)	NR
Carter et al. [57]	6	2	CT [AT + ST] (abdomen and back exercises; two medicine ball arm exercises from the supine position; rebound, static and dynamic balance exercises on a trampoline; bodyweight squatting exercise against a wall and stationary bicycle)	NR	NR	NR	NR
Gordon et al. [59]	4	5	AT (20–40 min of stationary bicycle, and treadmill)	NR	NR	40–60% (of reserve HR)	NR
	4	5	ST (16 exercises combine upper and lower body and core stability	10–15	1	NR	NR
Wunram et al. [60]	6	4	AT (Ergometer)	NR	NR		
	6	4	ST [VPT] (arm and shoulder contractions, trunk rotation, variety of leg and squat positions with 2–3 min for exercise and amplitude of 2 mm)	NR	NR	20 Hz	3

AT: aerobic training; CT: concurrent training; EG: experimental group; HR: heart rate; min: minutes; MR: maximum repetitions; NR: not reported; s: seconds; ST: strength training; S/w: session per week; VPT: vibration platform training; w: weeks.

## Data Availability

Not applicable.
